# Effectiveness of mindfulness meditation (Vipassana) in the management of chronic low back pain

**Published:** 2009-04

**Authors:** Sangram G Patil

**Affiliations:** Specialist Registrar in Anesthetics and ICM

**Keywords:** Mindfulness meditation, Chronic low back pain, Mindfulness based stress reduction

## Abstract

**Summary:**

Chronic low back pain (CLBP) is challenging to treat with its significant psychological and cognitive behavioural element involved. Mindfulness meditation helps alter the behavioural response in chronic pain situations. Significant body of research in the filed of mindfulness meditation comes from the work of Dr Kabat-Zinn. The current evidence in the field, though not grade one, shows that there is a place for mindfulness meditation in managing chronic pain conditions including CLBP. Further research to test the usefulness of mindfulness in CLBP should involve good quality randomized controlled trials of pure mindfulness based technique in matched subjects.

## Introduction

Chronic low back pain is challenging to treat with its significant economical burden, and very few effective treatment options. Psychological and cognitive factors are very important contributors to the pain intensity & disability associated with chronic pain. They may create a vicious cycle in which pain causes stress, and the stress, in turn, increases pain.

Various authorities have defined mindfulness in different ways, but the core of all of them is- mindfulness meditation is being alert and awake about the every present moment. The meditator concentrates on his/ her own breath in its entirety. Once he/ she becomes expert in it, he then starts body scan from head to toe and feels the body sensations with equanimous mind (non-reacting).

Kabat-Zinn^1^ has described the process of mindfulness meditation as learning to bear witness to one's own experiences without judgement. Thus mindfulness alters the behavioural pattern of the subject in different life situations, including chronic pain.

The aim of this review is to evaluate the available scientific evidence for effectiveness of mindfulness meditation in the management of CLBP.

## Literature review

There has been an extensive research in the field of management of CLBP. But meditation, in particular mindfulness meditation, as a tool to tackle CLBP associated problems has not been studied much. In this review, randomised clinical trials, systematic reviews, surveys and expert opinions have been examined for usefulness of mindfulness meditation for the management of CLBP.

## Mindfulness meditation & CLBP

Kabat-Zinn^2^ examined the effects of mindfulness based stress reduction (MBSR) program on 51 patients with chronic pain, and showed significant improvement on pain and psychological aspects, immediately on completion of the program. The subjects had chronic pain complaint of varied aetiology, including chronic low back pain. They had varying prior exposure to various medical and physical therapies for their chronic pain. All subjects (85% completed the sessions) were interviewed before and after completion of 10 week mindfulness meditation program; follow up questionnaire was sent at 2.5, 7 and 11 months post intervention. Each subject acted as his/her own control.

Follow up showed statistically significant progress towards greater energy, less pain and improved coping. The main limitations of the study were lack of control group, reliance on patient's subjective experiences for data collection, no observer blinding and non–rigorous selection process. But, this was a landmark study in the field of mindfulness meditation in chronic pain conditions. Being a nonrandomized, non blinded study it can not be accepted as very good research evidence, despite significant improvement in patients' condition.

In subsequent study Kabat-Zinn et al^3^ showed statistically significant reduction, as compared to control group, in measures of present moment pain, negative body image, inhibition of activity by pain. They also found positive effect on mood disturbance, and psychological symptoms, including anxiety and depression. Use of pain medications decreased and activity levels improved. Fifteen months follow up showed sustained improvement in all measures except the present moment pain. There was a high compliance to meditation practice in majority of cases. The controls used were chronic pain patients not involved in meditation program, but being treated with standard medical treatment. There was no randomization involved. Patients who met the criteria and agreed to dedicate to meditation schedules were enrolled into intervention group (selection bias). This was actually a descriptive comparative study of patients attending two different clinics. The study sample had twice the number of females as males. Males had more mood disturbances both pre and post intervention.

Kabat-Zinn et al^4^ found that mindfulness based meditation program had sustained positive effects in reduction in pain, psychological and overall health measures. Four year follow-up of 225 subjects showed high level of compliance with the meditation practices. The indices Body Parts Problem Assessment score (BPPA), Medical Symptom Check List(MSCL), Symptom Checklist-90-Revised(SCL-90-R), and Outcome Assessment score(OA) showed sustained improvement, which was statistically significant. Pain Rating Index (PRI) tended to reduce to pre-treatment level during follow-up. The shortcomings of this observational study were absence of control and possible bias due to self reporting questionnaires.

Berman and Singh^5^ onclude that subjects across various age groups and wide range of pain can be benefited from mind-body interventions which include educational lectures, mindfulness meditation and Qi Gong moment therapy. Their method involved three groups: mind-body intervention, physical therapy and standard physician led treatment. The study had a design flaws in patient selection and randomization. Due to patient willingness for not to go into particular group they had to create patient groups by matching the scores on the visual analogue scales (VAS). Also, the study results presented were mainly from mind-body intervention group- so can not really be compared with other two groups. The intervention group participants acted as control for themselves. The VAS showed positive changes in pain perception for all three questions, mood state, and functional ability. Beck Depression Scale showed significant improvement in eight of 21 subscales; coping strategy questionnaire showing the similar positive effects with regards to attitude towards pain and catastrophizing.

Wolf E et al^6^ state that patients suffering from CLBP improved significantly with breath therapy, which is a combination of mindfulness meditation, breathing and moments.

This was a randomized control trial in which participants were allocated into two groups: breath therapy and physical therapy. The measurements were done in a complex way (difficult to interpret easily). Baseline, six week and six months pain intensity (VAS), pain specific functional disability and overall health status (SF-36) were assessed. There was significant number of drop outs from the beginning, which has been described clearly in the study. The breath therapy group showed significant improvement over physical therapy in pain intensity, back pain related functional disability and emotional components of Short Form-36 score for functional health status(SF-36). The authors also conclude that breath therapy is safe in CLBP and it might teach coping skills and provide insight into the effects of stress on the body and low back pain.

M J Ott^7^, in her systematic review, states that mindfulness meditation is a skill that can be learned and, when practiced in a disciplined manner, is consistently effective in reducing stress and controlling pain. The key points in her review are as follows. MBSR is effective across cultures and economic levels. Mindfulness meditation has been shown to decrease symptoms of fibromyalgia, psoriasis, pain control among patients undergoing bone marrow transplant.

Plews-Ogan et al^8^ have found mindfulness meditation to be more effective and longer lasting for psychological improvements in patients with chronic musculoskeletal pain. Massage therapy is found to be better than MBSR in reducing chronic pain in these patients. They also conclude that MBSR is a feasible technique to be used in chronic pain patients.

The participants were randomized into three groups by computer generated randomization- standard care, mindfulness meditation (MBSR) and massage. The study was not observer blinded. There was also a high drop out rate amongst the MBSR arm subjects. None of the MBSR participants completed 8-weeks course. The pain outcome for MBSR was not significantly different from standard care. MBSR group had a statistically significant improvement in mental health scores over standard care group. Overall, this study was not very well conducted, so can not be graded as good evidence.

Carson et al^9^ report results of a pilot trial of 8-weeks loving kindness meditation in CLBP patients. Loving kindness meditation is a form of mindfulness meditation technique. This was a randomized controlled study of 43 participants with CLBP. They were randomized into two groups: loving kindness meditation intervention and standard care control. The intervention group underwent 8-weekly, 90-minute group sessions of loving kindness meditation. Two of 18 in intervention group and 4 of 25 in control group did not complete the study. Due to small sample size there was an insufficient power to this study. Despite this limitation, significant reductions in pain intensity, psychological distress, anxiety and hostility were observed in intervention group at the end of the study.

Morone et al^10^ state that eight mind-body interventions, looked at in their review, are feasible in an older population. Many of the therapies included modifications tailored for older adults. They have not found sufficient evidence for these eight mind-body interventions for reduction of chronic pain in older adults, though they mention the positive effects of mindfulness meditation in the studies by Kabat-Zinn^2^ and Morone et al^11^. The intervention they searched for are biofeedback, progressive muscle relaxation, transcendental meditation, mindfulness meditation, hypnosis, guided imagery, Qi Gong and yoga.

Roy C G^12^ has described a series of four case reports on relaxation techniques in chronic pain management, though none of them were actually CLBP. All four of them suffered from spinal cord trauma leading to paralysis of limbs associated with severe painful experiences. Simple meditation technique was taught to these patients and it improved their pain and mood status.

The author has concluded that relaxation techniques can aid in the rehabilitation of the patients with chronic pain in following ways. Minimum staff support is needed, usually just one therapist. Once the participants understand technique, they are encouraged to practice it independently. Meditation experience changes the attitude towards the pain. Thirdly, alteration of pain experience facilitates adjustment and rehabilitation, such as the partial relief from hopelessness and depression.

In a special communication of JAMA^13^, a 12 member NHS (UK) panel for technology assessment found strong evidence for the use of relaxation techniques in reducing chronic pain in variety of medical conditions. The panel has cited US agency for Health care Policy and Research (AHCPR, 1990) for its 4-point scale for the strength of evidence as strong, moderate, fair, or week. Relaxation techniques were found to have strong evidence in reducing chronic pain.

Morone et al^11^ state that only few alternatives exist for pain relief among older adults who have failed conventional therapy. They conducted a randomized control trial of mindfulness meditation program in old patients (>65 years age). They found a trend toward improvement in pain, pain related disability, global and physical health parameters. The McGill pain Questionnaire trended toward improvement but was not significant. No adverse events occurred during the intervention. When the intervention group was compared with the control for the above measures there was a trend toward improvement in all of them but this trend did not reach statistical significance.

## Practical issues with mindfulness meditation

### Feasibility in older population:

Morone et al^14^ conclude that 8-weeks mindfulness meditation program is feasible among community dwelling older adults with CLBP. They carried out the pilot feasibility randomized waiting list controlled trial of mindfulness meditation for CLBP. The intervention group received 8-weekly, 90-minute mindfulness meditation sessions and meditation homework. The control group patients were crossed over into meditation group immediately after the intervention group finished the program. The randomization and study design are of good quality. Thirteen out of 19 participants in meditation group completed the full 8 weeks program. Twelve of 18 from crossed over group completed the meditation program.

They followed the patients for three months and found sustained benefit from program as measured by continued meditation (MBSR) by the participants and improvement in physical function and pain acceptance. Nearly one half of the participants reported reduction in pain and sleep medications 3 months after completing the study. Overall, this is well-conducted study with good quality.

### Patient beliefs/acceptability:

Randolph el al^15^ found positive response to mindfulness based meditation interventions in American Christian population suffering from chronic pain from various medical conditions. They concluded that mindfulness meditation technique was relatively free of cultural effects of Buddhist teachings, or it was not offensive to Christians in their study population.

They carried out nonrandomized, prospective investigation in multidisciplinary chronic pain management clinic in West Texas. The patients initially had extensive psychological and medical assessment, during which, baseline data was gathered. As per their need, the patients then received combination of medical, physical and psychological therapies before participation in mindfulness training- Pain and Stress Management Program (P&SMP).

Most (95%) of the sample subjects reported to be affiliated with Christianity. The patients were allocated to P&SMP when they fulfilled the criteria and agreed to commit the time and efforts for mindfulness meditation. This means there was no strict selection criteria (selection bias). The study results showed significant improvement in sensory, affective and cognitive measures of chronic pain after 8-weeks mindfulness meditation program. The results were sustained at 1-year follow up. The authors also conclude that mindfulness meditation might act as a catalyst for positive response to medical treatment.

Sherman et al^16^ in their review have reported the results of telephonic interviews with the CLBP patients. The participants were asked, along with basic pain related questions, about willingness to try the complementary and alternative medicine (CAM) therapies at no extra cost and for $10 per visit co-pay. Respondents believed that meditation would be the least helpful of all the CAM therapies. Fewer respondents (27%) said they would be very likely to try meditation training if offered at no extra cost, as compared to acupuncture, chiropractic, massage and self-help back pain book (62%).

### Patient recruitment:

As mentioned above in the study by Berman and Singh^5^, some patients may be reluctant to participate in a study as a control or as a receiver of standard pain treatment. This creates a selection bias in a study. The controls can worsen during the period of study. They might seek other forms of therapies to control their symptoms, which might affect the study results.

### Intervener's approach:

The researchers may accept the technique of mindfulness meditation with different perspective, and this might over/under estimate the effects of the study involving mindfulness meditation.

Kwee et al^17^ explain that the researchers who have been involved in mindfulness based interventions can be grouped into two broad categories. First group views the expression ‘mindfulness-based’ in a ‘radical’ manner: i.e. in the actual sense of placing mindfulness at the very root and heart of therapeutic interventions, whenever founded on mindfulness (radicals). The second group seem to consider ‘mindfulness’ as a concept or a procedure that might be usefully incorporated in existing clinical protocols (incorporationists).

Kabat-Zinn^18^ states that it becomes critically important that those persons coming to the field with professional interest and enthusiasm recognize the unique qualities and characteristics of mindfulness as a meditative practice, with all that implies, so that mindfulness is not simply sized upon as the next promising cognitive behavioural technique or exercise, decontextualized, and “plugged” into a behaviourist paradigm with the aim of driving desirable changes, or of fixing what is broken.

### Delivery of mindfulness meditation technique:

The actual group intervention with mindfulness technique can meet with lots of challenges. The trainer competencies and beliefs may affect the progress of the participants significantly. Kabat-Zinn^19^ in his comments on ‘Majumdar et al: mindfulness meditation for health’, has mentioned- questions about curriculum and instructor competencies and qualifications are the important considerations in these studies. An intervention is ‘mindfulness base’ does not necessarily mean that it is, or it is as skilful or deep as may be required to be grounded in both the informal spirit and the formal practices of mindfulness.

### Complications of mindfulness meditation:

Apart from one patient in a study by Wolf E et al^6^, who became disturbed due to emergence of old memories, there are no other specific mindfulness related complications found in the literature. This study involved mindfulness based breath therapy and the patient dropped out of the study.

## Conclusion

The traditional pain therapies have their limitations and side effects. Mindfulness meditation is an inexpensive, non-invasive method which can be taught without any special equipment. There is definite evidence in favour of mindfulness meditation and its modifications in the management of CLBP. But we still need more good quality studies to justify the use of mindfulness meditation in CLBP patients on routine basis.

The future research should involve well designed randomised blinded control studies comparing CLBP patients in control group with standard therapy, against mindfulness meditation in intervention group. Obviously, it is not possible to conduct a double blind control trial due to the nature of the mindfulness intervention. Also, the structure of the meditation program needs a proper attention. It must be a pure mindfulness based program, rather than a combination of mind-body interventions. Most of the studies in this review involved combination of patients with chronic pain from various body parts, apart from CLBP. To be more specific to CLBP, the inclusion and exclusion criteria for study samples need to be strict.

## Implication for clinical practice

Though there is no grade 1 evidence for mindfulness meditation in the management of CLBP, it is quite cheap, harmless and still effective to certain extent in improving affective component of chronic pain. Also, it may lead to more pain acceptance, improved global health, and possibly improved compliance with current medical therapy.
